# Guideline-concordant antibiotic prescribing for community-acquired bacterial pneumonia (CABP) due to drug-resistant pathogens in the *All of Us* database

**DOI:** 10.1017/cts.2025.10143

**Published:** 2025-09-02

**Authors:** Corbyn M. Gilmore, Adriana Vargus, Grace C. Lee, Susanne Schmidt, Kelly R. Reveles, Carlos A. Alvarez, Christopher R. Frei

**Affiliations:** 1 College of Pharmacy, The University of Texas at Austin, San Antonio, TX, USA; 2 Pharmacotherapy Education and Research Center, Joe R. and Teresa Lozano Long School of Medicine, University of Texas Health Science Center at San Antonio, San Antonio, TX, USA; 3 Graduate School of Biomedical Sciences, University of Texas Health San Antonio, San Antonio, TX, USA; 4 South Texas Veterans Health Care System, San Antonio, TX, USA; 5 Department of Population Health Sciences, Joe R. and Teresa Lozano Long School of Medicine, University of Texas Health Science Center at San Antonio, San Antonio, TX, USA; 6 Texas Tech University Health Sciences Center, Jerry H. Hodge School of Pharmacy, Dallas, TX, USA; 7 Center of Excellence in Real-world Evidence, Texas Tech University Health Science Center, Dallas, TX, USA; 8 University Hospital, San Antonio, TX, USA; 9 School of Public Health, University of Texas Health Science Center at Houston, San Antonio Regional Campus, San Antonio, TX, USA

**Keywords:** Pneumonia, community-acquired infections, antibiotics, methicillin-resistant *Staphylococcus aureus*, *Pseudomonas aeruginosa*

## Abstract

**Introduction:**

Community-acquired bacterial pneumonia (CABP) contributes significantly to mortality and healthcare costs worldwide. The use of guideline-concordant antibiotic therapy for CABP is associated with improved outcomes.

**Methods:**

This was a retrospective cohort study of inpatients with CABP due to MRSA or *P. aeruginosa* in the *All of Us* database. The proportion of patients on guideline-concordant antibiotics or guideline-discordant antibiotics was compared within groups based upon patient age, sex, self-reported race, ethnicity, marital status, alcohol use, and tobacco use. Guideline concordance was determined using the 2019 IDSA/ATS CABP guidelines. Associations were further analyzed using multivariate logistic regression.

**Results:**

A total of 336 patients with CABP due to MRSA (152) or *P. aeruginosa* (184) were included. Guideline-concordant antibiotic therapy was prescribed to 70% of CABP-MRSA patients and for 57% of CABP-*P. aeruginosa* patients. Independently predictive factors of guideline-concordant antibiotic prescribing for CABP-*P. aeruginosa* patients were Non-Hispanic Black (NHB) vs. Non-Hispanic White (NHW) race (odds ratio = 0.30, 95% confidence interval = 0.12 – 0.75).

**Conclusion:**

In the *All of Us* database, the majority of CABP-MRSA and CABP-*P. aeruginosa* patients were prescribed guideline-concordant antibiotic therapy. Race was independently predictive of guideline-concordant antibiotic therapy for patients with CABP-*P. aeruginosa*, but not CABP-MRSA. NHB patients were less likely to receive guideline-concordant antibiotic therapy than NHW patients when treated for CABP-*P. aeruginosa.*

## Introduction

Antibiotics are one of the most widely prescribed medication classes and have been largely successful in treating bacterial infections. Antibiotics are prescribed to over 33% of patients treated in the outpatient setting and nearly 50% of patients treated in the inpatient setting [1]. However, disparities in antibiotic prescribing based on patient factors, such as race, ethnicity, age, or sex, have been documented in many settings and disease states [2,3]. These disparities in prescribing may lead to inequitable care, higher healthcare cost, and increased antimicrobial resistance [3–5]. The Centers for Disease Control and Prevention (CDC) estimate that antibiotic-resistant infections are responsible for more than 3 million infections and over 35,000 deaths in the United States, highlighting the serious threat posed by antimicrobial resistance [6]. The CDC and prior studies have classified Methicillin-resistant *Staphylococcus aureus* (MRSA) and *Pseudomonas aeruginosa* as leading causes of antibiotic-resistant infections, noting their significant contribution to hospital costs and mortality [6–11].

Community-acquired bacterial pneumonia (CABP) is a leading cause of inpatient mortality and high hospital costs [12–14]. For patients admitted to the hospital with CABP, the cost of treatment per patient is approximately $17,000 on average, with an average length of stay of 6 days and inpatient mortality exceeding 6% [14–16]. In 2019, the Infectious Disease Society of America (IDSA) and the American Thoracic Society (ATS) jointly published guidelines for the management of CABP, which outline recommendations for antibiotic therapy [17]. Guideline-concordant therapy is associated with improved outcomes for patients with CABP and is important for reducing antimicrobial resistance among causative pathogens [18–24].

While the use of and disparities in guideline-concordant therapy have been studied in CABP, no studies have focused on guideline-concordant therapy among CABP due to drug-resistant pathogens. Further, patients with CABP due to antibiotic-resistant infections, such as MRSA or *P. aeruginosa,* experience worse outcomes or may develop higher severity pneumonia (i.e., necrotizing pneumonia) [25,26]. Evaluating disparities in guideline-concordant antibiotic prescribing is important in identifying inequities in patient outcomes and care, which may inform the future development of targeted interventions that minimize these disparities. The objective of this study was to evaluate disparities in guideline-concordant antibiotic prescribing based on patient factors for patients admitted to the hospital with CABP due to MRSA or *P. aeruginosa.*

## Methods

### Study design and patient population

This was a retrospective cohort study of patients admitted to the hospital with a diagnosis of pneumonia due to MRSA or *P. aeruginosa* from 01/01/2011 to 10/01/2023, in version 8 of the *All of Us* database (cutoff date: 10/1/23). The screening dates for pneumonia due to MRSA or *P. aeruginosa* correspond to the publication year of the 2011 IDSA/ATS MRSA pneumonia treatment guidelines [27]. Patients were included in this study if they had a diagnosis of pneumonia (SNOMED 233604007) present in their record, were treated in the inpatient setting, and were prescribed antibiotics during the same encounter as the pneumonia diagnosis. Billing codes used to determine MRSA attribution were 124691000119101 (SNOMED), J15.212 (ICD-10), and 482.42 (ICD-9). Codes used to determine *P. aeruginosa* attribution were 41381004 (SNOMED), J15.1 (ICD-10), and 482.1 (ICD-9) [28,29]. Patients were excluded if they had documented causative pathogens other than MRSA or *P. aeruginosa* or if they had hospital-acquired pneumonia (HAP). For this study, HAP was defined as pneumonia with an initial diagnosis more than 48 hours after an inpatient admission. If a patient had multiple pneumonia visits on their record, only the earliest visit was counted.

### Data source

We used the controlled tier of version 8 of the *All of Us* database (released February 2025), which includes individual-level data from electronic health records (EHRs) and surveys collected through 10/01/2023. The *All of Us* research program is an initiative spearheaded by the National Institutes of Health (NIH) to increase the speed of medical research discoveries [30]. To date there are 861,000+ participants consented and 746,000+ enrolled [31]. Of those enrolled, 40% are from racial or ethnic minority groups, and many are from groups underrepresented in biomedical research [31]. Additionally, *All of Us* contains data on antibiotic prescribing and subtypes of pneumonia, making this study possible.

### Data collection

All tools used were provided in the cloud-based *All of Us* Researcher Workbench. Data were collected from the Controlled Access tier *All of Us* database using the cohort builder tool and dataset builder tool. Concept sets used for the dataset builder tool were “Pneumonia,” “Antibacterials for Systemic Use,” “Demographics,” and “The Basics Survey.” Data were then analyzed using Jupyter Notebook analysis environment and the R coding language inside the Researcher Workbench. Data were stored in the permanent storage area in the Researcher Workbench called the “Workspace Bucket,” which is a part of Google Cloud Storage.

### Study variables and end points

Patients were stratified into groups based on whether they received guideline-concordant antibiotic therapy or guideline-discordant antibiotic therapy. Anti-MRSA guideline-concordant antibiotic therapy was defined as vancomycin or linezolid [17,27,32]. Antipseudomonal guideline-concordant antibiotic therapy was defined as piperacillin-tazobactam, cefepime, ceftazidime, imipenem, meropenem, or aztreonam [17,32]. Guideline-discordant antibiotic therapy included any other antibiotics prescribed. Only antibiotics received within 48 hours of the pneumonia diagnosis were considered. If a patient received any guideline-concordant antibiotic therapy within 48 hours of the pneumonia diagnosis, then they were considered to have received guideline-concordant antibiotic therapy for CABP for the purpose of this study.

Demographic characteristics were determined using the prepackaged “Demographics” and “The Basics” concept set, which contains information on sociodemographic (age, sex, race, ethnicity) and behavioral (marital status, tobacco, and alcohol use) factors. Race, ethnicity, and behavioral characteristics were self-reported during the *All of Us* enrollment process [33]. Patients who reported their ethnicity as Hispanic in the Self-Reported Category column or Ethnicity column were classified as Hispanic. Patients who were not classified as Hispanic and reported their race as Black or White in the Self-Reported Category column or Race column were classified as Non-Hispanic Black (NHB) or Non-Hispanic White (NHW), respectively. All other patients who did not meet the above classification criteria for race and ethnicity were classified as Other due to the small sample size of other groups. The *All of Us* database provides patient-provided information on both gender identity and sex assigned at birth. For this study, we determined sex from the sex-assigned-at-birth question.

The primary outcomes for this study were receipt of anti-MRSA guideline-concordant antibiotic therapy or the receipt antipseudomonal guideline-concordant antibiotic therapy.

### Statistical analysis

Statistical comparisons were conducted in Jupyter Notebook inside the *All of Us* Researcher Workbench. Patient ages were compared using the Wilcoxon rank sum test. The chi-square test was used to compare dichotomous variables such as baseline characteristics. Counts of less than or equal to 20 are displayed as *n* ≤ 20 to comply with the *All of Us* data and statistic dissemination policy [34]. The actual count was used for statistical comparisons.

Multivariate logistic regression models were created with race and ethnicity (NHB vs. NHW, Hispanic vs. NHW, Hispanic vs. NHB) as the independent variable, receipt of either anti-MRSA guideline-concordant antibiotic therapy or antipseudomonal guideline-concordant antibiotic therapy as the dependent variable, and age (≥65 years vs. <65 years), sex (male vs. female), marital status (yes vs. no), alcohol use (yes vs. no), cigarette use (yes vs. no), and e-cigarette use (yes vs. no) as covariates. The second variable listed for each covariate serves as the reference variable for all regression models. Odds ratios and 95% confidence intervals for the receipt of anti-MRSA or antipseudomonal guideline-concordant antibiotic therapy were calculated using the outputs of the multivariate models. Patients who were classified as “Race and Ethnicity: Other” or did not provide an indication for any of the above factors were excluded from multivariable logistic regression analysis due to small sample size. The alpha level for significance was 0.05.

### Regulatory and ethics

All data collection, analysis, and dissemination were conducted in accordance with the *All of Us* Data User Code of Conduct, Data and Statistics Dissemination Policy, Publication and Presentation Policy, Policy on Stigmatizing Research, and all other policies therein [34–36]. Inclusion in this study was not based on age, sex, race, or ethnicity. Institutional Review Board approval was obtained from the University of Texas Health Science Center at San Antonio Institutional Review Board (IRB) and Office of Clinical Research (OCR) before beginning this study (Protocol number: 20220682EX).

## Results

**Figure 1.**
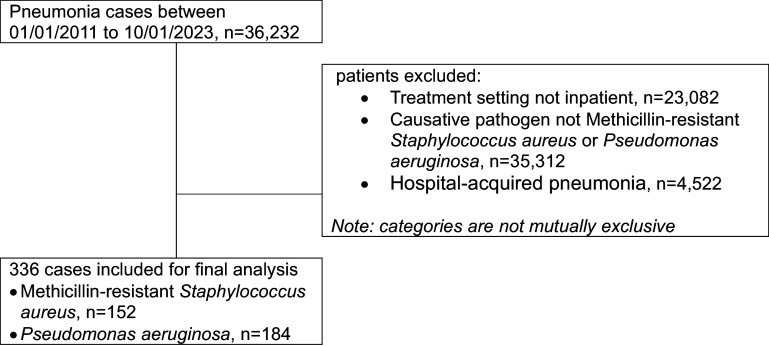
Study inclusion flowchart.

A total of 336 patients with CABP due to MRSA (152) or *P. aeruginosa* (184) met study criteria (Figure 1).

### Methicillin-Resistant *Staphylococcus aureus*

Guideline-concordant antibiotic therapy was prescribed to 70% of CABP-MRSA patients; all were prescribed vancomycin (98%) and/or linezolid (*n* ≤ 20). Other anti-MRSA agents prescribed include ceftroline (*n* ≤ 20) and tigecycline (*n* ≤ 20). No patients received tedizolid. We found no significant differences in baseline characteristics between the guideline-concordant and discordant groups (Table 1). There were no significant associations in the logistic regression analysis of patient factors and guideline-concordant anti-MRSA prescribing (Table 2).

**Table 1.** Baseline characteristics for CABP-MRSA patients prescribed guideline-concordant and discordant antibiotics

Characteristics	Guideline-Discordant, *n* = 46	Guideline-Concordant, *n* = 106	*P*-value
Age, median (IQR)	52.0(40.2 –65.0)	56.5(46.0 –65.0)	0.54
Race and Ethnicity	–	–	0.34
Non-Hispanic White	23 (50%)	53 (50%)	–
Non-Hispanic Black	*n*≤20	23 (22%)	–
Hispanic	*n*≤20	21 (20%)	–
Other	*n*≤20	*n* ≤ 20	–
Not Indicated	0	*n* ≤ 20	–
Sex	–	–	0.30
Female	22 (48%)	53 (50%)	–
Male	22 (48%)	52 (49%)	–
Intersex	*n*≤20	*n* ≤ 20	–
Not Indicated	*n*≤20	*n* ≤ 20	–
Marital Status	–	–	0.61
Divorced	*n* ≤20	*n* ≤ 20	–
Married	*n* ≤ 20	25 (24%)	–
Living with Partner	*n* ≤ 20	*n* ≤ 20	
Never Married	*n* ≤ 20	36 (34%)	–
Separated	*n* ≤ 20	*n* ≤ 20	–
Widowed	*n* ≤ 20	*n* ≤ 20	–
Not Indicated	*n* ≤ 20	*n* ≤ 20	–
Alcohol Use	–	–	0.31
Alcohol Use: Yes	41 (89%)	88 (83%)	–
Alcohol Use: No	*n* ≤ 20	*n* ≤ 20	–
Not Indicated	*n* ≤ 20	*n* ≤ 20	–
Smoking Status	–	–	0.06
100 Cigs Lifetime: Yes	24 (52%)	63 (59%)	–
100 Cigs Lifetime: No	*n* ≤ 20	43 (41%)	–
Not Indicated	*n* ≤ 20	0	–
Electronic Smoke Participant	–	–	0.07
Electronic Smoke Participant: Yes	*n* ≤ 20	*n* ≤ 20	–
Electronic Smoke Participant: No	39 (85%)	86 (81%)	–
Not Indicated	*n* ≤ 20	0	–

Bold italics indicates statistically significant findings.

Counts of less than or equal to 20 are displayed as *n*≤20 to comply with the *All of Us* data and statistic dissemination policy [34].

The actual n was used for statistical comparisons. CABP: Community-acquired bacterial pneumonia. MRSA: Methicillin-resistant *Staphylococcus aureus*.

**Table 2.** Logistic regression analysis of patient factors and guideline-concordant anti-MRSA prescribing

Characteristics	Estimate	OR	95% CI	*P*-value
Model 1: MRSA				
Age (≥65 vs. <65)	0.24 ± 0.48	1.27	0.51 – 3.36	0.62
NHB vs. NHW	0.49 ± 0.60	1.64	0.54 – 5.74	0.41
Hispanic vs. NHW	−0.18 ± 0.52	0.83	0.30 – 2.39	0.72
Hispanic vs. NHB	−0.68 ± 0.68	0.51	0.13 – 1.90	0.32
Sex (Male vs. Female)	0.24 ± 0.43	1.27	0.55 – 2.99	0.58
Married (Yes vs. No)	−0.41 ± 0.43	0.66	0.28 – 1.58	0.34
Cigarette Use (Yes vs. No)	−0.08 ± 0.46	0.92	0.37 –2.25	0.85
Alcohol Use (Yes vs. No)	−0.38 ± 0.64	0.69	0.17 – 2.25	0.55
Electronic-Cigarette Use (Yes vs. No)	0.50 ± 0.65	1.65	0.49 – 6.65	0.44

Bold italics indicates statistically significant findings.

Patients who were classified as Race and Ethnicity: Other or did not provide an indication for any of the above categories were excluded from logistic regression of Patient Factors and Guideline-Concordant Anti-MRSA Prescribing (total *n* = 23) due to small sample size.

The second variable listed is the reference group used in this model.

MRSA: Methicillin-resistant *Staphylococcus aureus,* NHB: Non-Hispanic Black, NHW: Non-Hispanic White.

### Pseudomonas aeruginosa

Guideline-concordant antibiotic therapy was prescribed to 57% CABP-*P. aeruginosa* patients; all were prescribed cefepime (33%), piperacillin-tazobactam (21%), ceftazidime (*n* ≤ 20), meropenem (*n* ≤ 20), aztreonam (*n* ≤ 20), and/or imipenem (*n* ≤ 20). Guideline-concordant antibiotic therapy patients had fewer NHB patients (*p* = 0.04) (Table 3). In the multivariate logistic regression analysis of patient factors and guideline-concordant antipseudomonal prescribing, NHB patients were less likely to receive guideline-concordant antibiotic therapy when compared to NHW patients (OR 0.30, 95% CI 0.12 –0.75) (Table 4).

**Table 3.** Baseline characteristics for CABP-*Pseudomonas aeruginosa* patients prescribed guideline-concordant and discordant antibiotics

Characteristics	Guideline-Discordant, *n* = 80	Guideline-Concordant, *n* = 104	*P*-value
Age, median (IQR)	60.0 (48.0 – 67.0)	57.5(48.0 – 70.2)	0.40
Race and Ethnicity	–	–	***0.04***
Non-Hispanic White	38 (48%)	66 (64%)	–
Non-Hispanic Black	*n* ≤ 20	*n* ≤ 20	–
Hispanic	*n* ≤ 20	*n* ≤ 20	–
Other	*n* ≤ 20	*n* ≤ 20	–
Not Indicated	*n* ≤ 20	*n* ≤ 20	–
Sex	–	–	0.37
Female	37 (46%)	55 (53%)	–
Male	42 (53%)	49 (47%)	–
Intersex	*n* ≤ 20	0	–
Not Indicated	*n* ≤ 20	0	–
Marital Status	–	–	0.15
Married	28 (35%)	39 (38%)	–
Divorced	*n* ≤ 20	*n* ≤ 20	–
Living with Partner	*n* ≤ 20	*n* ≤ 20	–
Never Married	*n* ≤ 20	24 (23%)	–
Separated	*n*≤20	*n* ≤ 20	–
Widowed	*n* ≤ 20	*n* ≤ 20	–
Not Indicated	*n* ≤ 20	0	–
Alcohol Use	–	–	1.0
Alcohol Use: Yes	69 (87%)	89 (86%)	–
Alcohol Use: No	*n* ≤ 20	*n* ≤ 20	–
Not Indicated	*n* ≤ 20	*n* ≤ 20	–
Smoking Status	–	–	0.31
100 Cigs Lifetime: Yes	43 (54%)	45 (43%)	–
100 Cigs Lifetime: No or Not Indicated	37 (44%)	59 (57%)	–
Electronic Smoke Participant	–	–	0.20
Electronic Smoke Participant: Yes	*n* ≤ 20	*n* ≤ 20	–
Electronic Smoke Participant: No	68 (85%)	95 (91%)	–
Not Indicated	*n* ≤ 20	*n* ≤ 20	–

Bold italics indicates statistically significant findings.

Counts of less than or equal to 20 are displayed as *n* ≤ 20 to comply with the *All of Us* data and statistic dissemination policy [34].

The actual *n* was used for statistical comparisons. CABP: Community-acquired bacterial pneumonia.

**Table 4.** Logistic regression analysis of patient factors and guideline-concordant antipseudomonal prescribing

Characteristics	Estimate	OR	95% CI	*P*-value
Model 2: *P. aeruginosa*				
Age (≥65 vs. <65)	−0.24 ± 0.38	0.78	0.37 – 1.65	0.52
NHB vs. NHW	***−1.20 ± 0.47***	***0.30***	***0.12 – 0.75***	***0.01***
Hispanic vs. NHW	−0.10 ± 0.46	0.90	0.37 – 2.24	0.82
Hispanic vs. NHB	1.09 ± 0.56	2.99	1.01 – 9.25	0.05
Sex (Male vs. Female)	−0.31 ± 0.36	0.73	0.36 – 1.47	0.39
Married (Yes vs. No)	0.17 ± 0.36	1.18	0.59 – 2.38	0.64
Cigarette Use (Yes vs. No)	−0.45 ± 0.38	0.64	0.30 – 1.35	0.24
Alcohol Use (Yes vs. No)	0.16 ± 0.60	1.17	0.35 – 3.81	0.79
Electronic-Cigarette Use (Yes vs. No)	−0.52 ± 0.61	0.60	0.18 – 1.94	0.39

Bold italics indicates statistically significant findings.

Patients who were classified as Race and Ethnicity: Other or did not provide an indication for any of the above categories were excluded from logistic regression of Patient Factors and Guideline-Concordant Antipseudomonal Prescribing (total *n* = 23) due to small sample size.

The second variable listed is the reference group used in this model.

*P. aeruginosa: Pseudomonas aeruginosa.* NHB: Non-Hispanic Black. NHW: Non-Hispanic White.

## Discussion

The present study was the first to evaluate antibiotic prescribing trends for inpatients with CABP due to MRSA or *P. aeruginosa* in the *All of Us* database. Of the patients admitted to the hospital with CABP due to MRSA, nearly three-quarters (70%) were prescribed guideline-concordant antibiotic therapy with no observed differences based on patient factors. For patients admitted to the hospital due to CABP due to *P.* aeruginosa, 57% received guideline-concordant antibiotic therapy and NHB patients were less likely to receive antipseudomonal guideline-concordant antibiotics than NHW patients.

### Health disparity in pneumonia

Previously, our research team investigated differences in guideline-concordant antibiotic prescribing for patients admitted to the hospital with general CABP in the *All of Us* database. Of the 1,608 patients included, less than half (45%) received guideline-concordant antibiotic therapy for CABP [37]. Further, we found that NHB patients were 36% less likely than NHW patients, and Hispanic patients were 34% more likely than NHW patients to receive guideline-concordant antibiotic therapy for the treatment of CABP [37]. Therefore, our findings in these two studies are consistent in a mixed CABP population (all pathogens) and a CABP-*P. aeruginosa* population.

In addition to the above study, a study of 5,820 inpatients with pneumonia by Evans et al. identified disparities based on patient race and ethnicity [28]. They found that younger non-Hispanic Black patients were less likely to receive anti-pseudomonal antibiotics than any other race group considered [28]. They also found no difference in receipt of anti-MRSA therapy by race [28]. Both of these findings are consistent with our results. Other studies of general CABP report no difference by race or ethnicity in receipt of guideline-concordant antibiotic therapy, both in the general inpatient ward and ICU, which is different than our results [38,39]. Direct comparison between these studies and ours are difficult due to differences in patient populations and topics. Nevertheless, both studies still report other variations in care and health outcomes in CABP treatment that are based on patient factors, which is consistent with our findings [38,39].

### Health disparity in other disease states

Disparity in antibiotic prescribing based upon patient factors has been well documented in settings other than pneumonia [2,3]. In one scoping review of health equity and antibiotic prescribing in the United States from 2000–2022, Kim et al. found that Black patients were less likely to receive antibiotics when compared to White patients, which is consistent with our results [3]. In a study evaluating health disparity in the ambulatory care setting, Young et al. found that Black and Hispanic patients had the highest rates of overall antibiotic prescribing and inappropriate antibiotic prescribing, and that the impact of patient factors on antibiotic prescribing may be additive [2]. The cause of these disparities remains unclear, but it is likely multifactorial. Additional studies on the drivers of health inequity are needed.

### Strengths

CABP is a significant contributor to inpatient mortality and rising hospital costs worldwide. The selection of proper antibiotic therapy, which is essential for improving clinical outcomes, is a complex issue further complicated by the emergence of drug-resistant pathogens. Evidence regarding guideline-concordant antibiotic prescribing for CABP due to drug-resistant pathogens like MRSA or *P. aeruginosa* is extremely limited, even more so for health disparity in prescribing practices. The present study is the first to evaluate guideline-concordant prescribing for inpatients with CABP due to MRSA or *P. aeruginosa.* Furthermore, clinical practice guidelines are often used to develop hospital policies and workflows. Evaluation of current guidelines and their use is necessary to ensure timely administration of therapy, selection of effective therapy, lower healthcare costs, and improve patient outcomes. Identification of differences in prescribing practices is the first step in understanding them and generating future intervention-based studies investigating health disparity, novel treatment strategies, and optimization of current treatment delivery algorithms.

### Limitations

Due to its observational design, this study has some potential limitations, including limited generalizability to the entirety of the US, lack of local microbiological data, lack of disease severity data, inability to display counts of ≤20, inability to control for variations in decision workflows between institutions and individual providers, inability to evaluate health outcomes, and potential selection bias due to nonrandomization of study arms. Additionally, this study was subject to a small sample size due to specific inclusion criteria and reliance on billing codes of documented causative pathogens. Thus, it may contain potential type 2 errors in both the MRSA and *P. aeruginosa* populations and may be underpowered. To limit study inclusion to only patients with CABP, rather than HAP, only pneumonia cases diagnosed within the first 48 hours of an inpatient admission were included; however, some HAP patients might have still been included.

To overcome the lack of local microbiological data, this study only considered pneumonia cases that had been attributed to MRSA or *P. aeruginosa* and coded as such using SNOMED, ICD-9, or ICD-10 codes. However, using pathogen-specific billing codes likely underestimated the true prevalence of these pathogens and may have introduced selection bias, as cases might be from providers who specialized in these pathogens. Further, pathogen-specific billing codes are rarely used in practice [40,41]. One large cross-sectional study of patients hospitalized with pneumonia found that pathogen-specific ICD-9 codes appear to have limited sensitivity but high specificity [40]. Another study examining the validity using ICD-10 codes to identify hospitalized pneumonia found that ICD-10 codes are useful for identifying all-cause pneumonia but less helpful in identifying subtypes of pneumonia [41].

To ensure adequate analysis of prescribed antibiotics, all inpatient antibiotics prescribed within 48 hours of the pneumonia diagnosis were included in this study; however, duration of therapy was not included in this study due to incompleteness of duration data. Additionally, the definition of guideline-concordant used in this study was based on the receipt of at least one dose guideline-concordant antibiotics in the first 48 hours. However, we did not reclassify guideline-concordant patients if they also received guideline-discordant antibiotic therapy at any time, which may contribute to underestimation of guideline-discordant prescribing. Despite these limitations, this work provides valuable insights into prescribing practices for CABP due to MRSA and *P. aeruginosa* and can inform future intervention-based studies.

## Conclusions

In the *All of Us* database, 70% of CABP-MRSA and 57% of CABP-*P. aeruginosa* patients were prescribed guideline-concordant antibiotic therapy. Cases were defined using billing codes of documented causative pathogens. Race was independently predictive of guideline-concordant antibiotic therapy for patients with CABP-*P. aeruginosa*, but not CABP-MRSA. NHB patients with CABP due to *P. aeruginosa* were less likely to be prescribed guideline-concordant antibiotic therapy when compared to NHW inpatients. Evaluation of the implementation of current guidelines is necessary to ensure timely administration of effective therapy and improve patient outcomes. Variations of care and health disparity in guideline-concordant antibiotic therapy prescribing for CABP due to drug-resistant pathogens lead to suboptimal patient outcomes. Identification of these disparities is essential in minimizing them and promoting health equity for all patients.
